# Editorial: Recent advances in photokinetics

**DOI:** 10.3389/fchem.2024.1474428

**Published:** 2024-08-23

**Authors:** Mounir Maafi

**Affiliations:** Leicester School of Pharmacy, De Montfort University, Leicester, United Kingdom

**Keywords:** photokinetics, quantum yield, photochemistry, monochromatic/polychromatic light, Π-order kinetics

Light processes have been increasingly involved in modern research, extending to a wide selection of fields in chemistry and biology. The trend is sustained by economic, environmental, and technological needs and advances. In this context, harvesting solar light has directed research options toward replacing artificial lamps, which historically were employed for the investigation and application of photoactive species sensitive to ultraviolet and/or visible electromagnetic radiation. In parallel to this goal, research in the area of photoinduced reactions targeted the standardization of metrics. Indeed, despite photochemistry being almost a 150-year-old subject, the investigative tools available to photo-kineticists and researchers interested in evaluating the parameters of photoreactions are still limited in number and performance.

The Research Topic, “Recent advances in photokinetics,” offers a forum for discussion of the achieved progress and the latest tools proposed in photokinetics (i.e., the kinetics of photoreactions). The relevance of this Research Topic stems from the lack of documentation on this specific area of photochemistry. A deficiency indicated by the fact that only a single book that is fully dedicated to photokinetics exists in the literature (Mauser, 1998). Several chapters in various books have attempted to shed light on aspects of the quantification of photoreactivity, but only focusing on particular photosystems (Brown, 1971; Crano and Guglielmetti, 1999; Bouas-Laurent and Durr, 2001; Tonnesen, 2004; Griesbeck et al., 2012; Nakatani et al., 2016; Pianowski, 2022).

In this context, it is striking to observe that the order of a photoreaction is not yet known, and the community has not reached a consensus on standard photoreaction metrics. Perhaps the most compelling aspect of such a situation is the current and common usage of purely thermal reaction kinetic models, such as zeroth, first, and second-order kinetics, for the treatment of photokinetic data (Crano and Guglielmetti, 1999; Tonnesen, 2004). Not only does that strategy hinder the development of adequate methods for the investigation of photoreactions, but also, the use of thermal kinetic models is practically inappropriate, both in performance and precision, for assessing photochemical events and quantities.

The papers included in this Research Topic, albeit few, nonetheless offer a substantial sample of information on the subject. 

Sunlight-based photocatalysis for the purpose of simultaneously degrading a wide variety of pollutants from polluted water sources has been reviewed (John et al.). The targeted technology employs photocatalytic fuel cells (PFCs) that combine the production of electric power and photocatalytic degradation of pollutants, where both processes are triggered by exposure to renewable sunlight. The development of PFCs has followed different strategies aimed at technically upgrading conventional photoelectrochemical cell designs to be advantageously exploited in PFCs and fostering research on the design and development of materials/composites for photosensitive electrodes. The photoexcited electron–hole pairs generated upon light irradiation in PFCs induce electric energy in the external circuit, with the photogenerated holes in the anode compartment causing the degradation of fuel through the conversion of hydroxyl ions into hydroxyl radicals (one of the most reactive oxygen species), whereas the photoproduced electrons contributed to hydrogen evolution reactions at the cathode. The reaction and kinetics within these basic and more elaborate designs have been laid out together with examples of organic pollutants degradation vs. irradiation time plots, current density–voltage characteristic curves, and irradiance-time profiles. The applications to the generation of hydrogen, reduction of either CO_2_ or heavy metals, hydrogen peroxide production, TOC and total nitrogen removal, and sensory devices were also discussed in relation to various types of PFCs.

An illustration of a biological photosystem (Yu et al.) was provided by the improvement of both the photothermal conversion efficiency and the fluorescence quantum yield, recorded for a novel multifunctional probe (Gd-EB-ICG, or GI) self-assembled with endogenous albumin into drug–albumin complexes (GIAs). This rather simple strategy resulted in a synergistic effect for fluorescence/magnetic resonance dual-modal imaging and photothermal therapy, providing an all-new light for multifunctional probes that could be translated into clinical applications. The method is expected to provide accurate tumor visualization to guide their photothermal therapy and might represent a breakthrough in cancer treatment.

Novel fundamental photokinetic concepts and tools have been introduced in two articles dealing with irradiation using mono- (Maafi) and polychromatic (Maafi) lights. A general formula for the integrated rate law was proposed and tested against fourth-order Runge–Kutta numerical integration traces. This formula is the first of its kind in photochemistry literature, applies to any photosystem, and proves that photoreactions obey Φ -order kinetics. The latter, being different from the thermal kinetic models, provides, together with the general formula, an efficient tool to quantify and readily fit photokinetic traces of photosystems. Among the prominent findings of these studies, a general formula was derived for the quantum yield of the reactant subjected to monochromatic light irrespective of whether or not the mechanism of the reaction and/or the absorptivities of its reactive species are known, offering, therefore, the possibility to investigate the effect of the monochromatic irradiation wavelength on the reactant quantum yield (Maafi, 2024). An elucidation procedure and an actinometric standardization method were also presented in detail, respectively, for solving the photokinetics for the unknown intrinsic reaction parameters and to transform any system into a reliable actinometer even when the mechanism of this system and the intrinsic parameters of the reaction are not available. An equivalent actinometric procedure was also proposed for photoreactions under polychromatic light (Maafi). These findings represent a paradigm shift in photokinetics, allowing a rationalization of the kinetic behavior of photoreactions within a framework agreeing with the fundamental equations that describe the physical system at hand. This research is thought to contribute to developing a more precise photokinetic set of investigative tools. 
